# The War Calls

**Published:** 1918-08

**Authors:** 


### The War Calls.

The war is callling for big men in every walk of life. They are not only needed in the service abroad, but are needed just as much here at home. The call comes especially strong to medical men and thousands of them have responded to the appeal to go abroad. Those who are left behind are expected to conduct themselves in such a way that the professional standards will not be lowered. Naturally the call to serve abroad has secured the men of middle life, those right in the vigor of manhood, men of experience who have lost none of their interest in their life work and who are capacitated for doing big things in a big way. Perhaps the majority of those who are left behind are either men who are immature and without experience or those who by reason of their age are thought not to be sufficiently virile to measure up to all the requirements. To these of course are added those who are physically weak and those whose family encumbrances and responsibilities have prevented them from taking up duties abroad.

It devolves on those who are left behind to show that they are fully able to keep up the professional standard, and therefore, they must be first of all students. If they have been inclined to leave the study of progressive medicine to the younger men, they should again take up the responsibility of keeping up with the advances made in medicine. They do not have the younger men here now to counsel with and therefore they must know all that they and the younger men have together known. To do this they must study just as they did in their younger days. Of course many of them have always done this, but some have not. In war times there must be no slackers, for all that is within the power of men to do must be done to win in this fight, and to win the public must be kept in good health.

It is the duty of those left behind to be liberal in their relations with their fellow men, to be broad in conceding to others the right to their own opinions and practices, yet zealous in defending one's own views. Narrowness, intolerance and jealousy should be wiped out. The man who is struggling to benefit others should be encouraged and not hindered and that, too, despite the fact that you may not be able to agree with them in their methods. All the good that others can do should be recognized even though it may not be accomplished just in the way you would have done it.

The war is a great leveler of men and of ideas. It drives the world away from many preconceived ideas and makes the public more liberal in its estimate of others, for it brings every one of sense and brains to a recognition that after all what the world knows is very little and inconsequential and that a thing accepted as a boon today may be regarded as a curse tomorrow.

The practice of surgery especially is undergoing a great change, one that a few years ago was as little dreamed of as a successful flying machine, and who can guess just the place that surgery and medicine may hold in the world when the great contest is over?

Specialization will in all probability come more and more into vogue, and for every part of the body there may be a special practitioner. The world is beginning to concede that one little brain cannot know all about everything or about every part of the body. Only a few years ago the medical profession was inclined to frown upon the specialist who gave his entire time to the eye, the throat or any other special part of the body, at least to the exent of refusing to recognize such specialists as physicians. By many regular physicians they were regarded somewhat as fakirs. Now the eye, ear, nose, throat, lung, stomach and other classes of specialists are recognized as experts by all physicians, and the old-fashioned family physicians who were equally adept in treating all parts of the body have almost become obsolete.

The sanitary specialist is making a place for himself in medicine, and some think that the medical men of the future will be largely engaged in preventive medicine—in keeping people well, instead of in curing them after they become sick.

So, be liberal; the branch of medical practice or the methods you are condemning today may have the upperhand tomorrow, and them who are adhering to the old ideas and practices may be relegated to the professional ash-heap. Above all, be charitable with the other fellow's views and methods; after all he may be right, and you may just have failed to keep step with him. In many cases the war is likely to show that many who have been regarding themselves as progressive, may have been making progress in the wrong way—going backward. Keep your ears open for the war's call.

---

He Knew.—A little boy asked his father, a confirmed dyspeptic:

"Dad, did Moses suffer with indigestion?"

"I am sure I don't know," snapped his father, whose temper was rather soured by his infirmity.

"Well, I think he must have had it, for our teacher told us on Sunday that God gave him two tablets."—*The University Gazette.*

